# Mite species inhabiting commercial bumblebee (*Bombus terrestris*) nests in Polish greenhouses

**DOI:** 10.1007/s10493-012-9510-8

**Published:** 2012-01-24

**Authors:** Elżbieta Rożej, Wojciech Witaliński, Hajnalka Szentgyörgyi, Marta Wantuch, Dawid Moroń, Michal Woyciechowski

**Affiliations:** 1 Institute of Environmental Sciences, Jagiellonian University, ul. Gronostajowa 7, 30-387 Kraków, Poland; 2 Department of Comparative Anatomy, Institute of Zoology, Jagiellonian University, Gronostajowa 9, 30-387 Kraków, Poland; 3 Institute of Systematics and Evolution of Animals, Polish Academy of Sciences, Sławkowska 17, 31-016 Kraków, Poland

**Keywords:** *Bombus terrestris*, Acaridae, Laelapidae, Parasitidae, Invasive species, Commensal species

## Abstract

Nests of social insects are usually inhabited by various mite species that feed on pollen, other micro-arthropods or are parasitic. Well-known negative effects of worldwide economic importance are caused by mites parasitizing honeybee colonies. Lately, attention has focused on the endoparasitic mite *Locustacarus buchneri* that has been found in commercial bumblebees. However, little is known of other mites associated with commercial bumblebee nests. Transportation of commercial bumblebee colonies with unwanted residents may introduce foreign mite species to new localities. In this study, we assessed the prevalence and species composition of mites associated with commercial bumblebee nests and determined if the mites are foreign species for Poland and for Europe. The study was conducted on 37 commercial bumblebee nests from two companies (Dutch and Israeli), originating from two greenhouses in southern Poland, and on 20 commercial bumblebee colonies obtained directly from suppliers. The species composition and abundance of mites inhabiting commercial bumblebee nests were determined. Seven mite species from three families were found in nests after greenhouse exploitation. The predominant mite species was *Tyrophagus putrescentiae* (Acaridae) that was a 100-fold more numerous than representatives of the family Laelapidae (*Hypoaspis marginepilosa, H. hyatti, H. bombicolens*). Representatives of Parasitidae (*Parasitellus fucorum, P. crinitus, P. ignotus*) were least numerous*.* All identified mite species are common throughout Europe, foreign species were not found. Mites were not detected in nests obtained directly from suppliers. We conclude that probably bumblebee nests are invaded by local mite species during greenhouse exploitation.

## Introduction

Social and solitary bees are hosts to a wide diversity of mites (Eickwort 1994; Klimov et al. 2007; Park et al. 2009). Acarine parasites of honeybees such as *Acarapis woodi* and *Varroa destructor* have been intensively studied as they are problematic pests that influence beekeeping and pollination services at a global scale (Sammataro et al. 2000; Baker et al. 2005; Baker 2010). Lately, more attention has been paid to bumblebee mite associates. Commercial bumblebee rearing started over 20 years ago and nowadays is a global business (Velthuis and Van Doorn 2006). The rearing of bumblebee colonies at high densities in commercial companies provides an opportunity for various pathogens to develop and reach a higher prevalence than their counterparts in natural populations (Colla et al. 2006; Otterstatter and Thomson 2008). Moreover, European greenhouses import bumblebee colonies from outside of Europe (Velthuis and Van Doorn 2006) which may facilitate the transportation of alien bumblebee-associated mites as well as other parasites and pathogens. Research on bumblebee colonies has concentrated mainly on protozoan parasites, their effect on host fitness and possible treatments (Imhoof and Schmid-Hempel 1999; Whittington and Winston 2003; Rutrecht and Brown 2007; Otti and Schmid-Hempel 2008). Some studies have detected protozoan parasites in the Canadian greenhouse bumblebee, *Bombus occidentalis* (Whittington and Winston 2003), and European commercial colonies of *Bombus terrestris* (Niwa et al. 2004). Others have shown that commercially bred bumblebees can pose a threat to local populations as vectors transmitting the protozoan parasites *Nosema bombi* and *Crithidia bombi* (Colla et al. 2006).

Another example of a parasite disseminated with commercial bumblebee colonies is the endoparasitic mite *Locustacarus buchneri*. The mite feeds and reproduces mainly in the abdominal air sacks of adult queens and worker bumblebees (Yoneda et al. 2008b). Individuals of this species, with a foreign genome, were found both among commercial colonies exported from Europe to Japan and among Japanese native bumblebee *Bombus hypocrita* (Goka et al. 2001, 2006, Yoneda et al. 2008a, b). Therefore this parasitic mite has been known from commercial bumblebees for a decade. This tracheal mite is known to attack at least 25 wild bumblebee species across the Holarctic region (Husband and Husband 1997). *Locustacarus buchneri* was also detected among commercial colonies of *B. terrestris* and *Bombus ignitus* imported from the Netherlands and Belgium (Goka et al. 2006). Although little is known about the influence of mites on bumblebees, it is considered as negative. Parasitized bumblebees have shorter lifespans than unparasitized bees (Otterstatter and Whidden 2004). Transportation of *L. buchneri* with commercial bumblebees from Europe to Japan exemplifies a potential route for other unwanted bumblebee associates that in the same way can spread to new localities. Therefore studies detecting other species related to commercial bumblebee nests are needed.

There are other mites associated with bumblebees that live and reproduce in bumblebee nests on resources such as nectar and pollen (Schmid-Hempel 1998), but have never been studied in commercial bumblebee nests. These mites presumably do not have a strong positive or negative effect on their host, as they are saprophagous or mostly fungivorous and a social insect nest is one of many potential habitats. They are not exclusively dependent upon nourishment provided in the nest (Eickwort 1994; Schmid-Hempel 1998). Some bumblebee-associated mites recorded from wild bumblebees are phoretic (Schwarz et al. 1996; Schwarz and Huck 1997; Huck et al. 1998; Koulianos and Schwarz 1999; Chmielewski and Baker 2008), using bumblebees for transport. In such cases, the mite and host species exhibit synchronization of life cycles. Bumblebees form annual colonies and only the young queens overwinter. Therefore, mites must attach themselves to new queens in autumn and accompany them to hibernation sites (Eickwort 1994; Schwarz et al. 1996; Huck et al. 1998). When an overwintered queen founds a new colony in the spring, the mites detach and colonize the new nest (Goulson 2003). Four to six mite species may be found on an overwintering queen and more than 20 mite individuals may be present on one bumblebee (Schwarz et al. 1996).

Commercialization of bumblebees brings new opportunity for associated mite species to reproduce at high rates under favourable conditions, as commercial colonies are supplied with sugar solution and pollen (Velthuis and Van Doorn 2006). There is an abundance of food for saprophagic mites that can, in turn, be a food source for predatory mites. In greenhouses that use bumblebee colonies, mites have ideal conditions for transfer between colonies due to the high densities of pollinators and their nests. Additionally, stable and relatively high humidity and temperature are ensured by bumblebee nests (Velthuis and Van Doorn 2006) and the greenhouse itself. The sale of commercial colonies beyond the natural distribution of bumblebee subspecies (Velthuis and Van Doorn 2006) enables associated mites to expand their distributions and settle new localities. As large scale exportation of bumblebee colonies takes place worldwide (Velthuis and Van Doorn 2006), the introduction of mite species (and other bumblebee-associated organisms) into areas formerly unoccupied becomes likely.

In this study we investigated the species composition and frequency of mites in commercial bumblebee (*B. terrestris*) nests before and after exploitation in greenhouses. We paid particular attention to the detection of foreign (i.e. Middle Eastern) mite species because one of the suppliers originates from Israel. The first experiment was performed on nests after greenhouse use in order to obtain information on species composition and abundance of mites inhabiting commercial nests that were exposed in greenhouses. In this experiment we also examined how the number of mites inhabiting bumblebee nests changes with time spent in greenhouses. The second experiment was performed on nests before greenhouse use to check whether nests are already mite-infested directly from the suppliers, followed by an assessment of species composition and abundance.

## Methods

The methods are described in chronological order, according to availability of studied material and performed experiments. First, we investigated commercial nests after greenhouse use, and afterwards nests obtained directly from suppliers (without exposition in greenhouses).

### Commercial bumblebee nests after exploitation in greenhouses

These nests were studied after eight or more weeks of greenhouse exploitation (depending on greenhouse management). Nests were examined in the laboratory for mite presence and species composition. This part of the study was carried out in September 2006. We obtained 37 nests from two greenhouses in southern Poland, Sarnów—22 nests (11 nests from Israeli and 11 from Dutch suppliers), and Krzeszowice—15 nests (five nests from Israel and 10 from the Netherlands). Both greenhouse complexes have existed for nearly 35 years and have used bumblebee pollination from the early 1990’s. The examined nests contained live bumblebees and differed in time (8–18 weeks) spent in greenhouses. Bumblebees were used for the pollination of tomato crops.

Nests were removed from plastic boxes in which they are normally sold. Next, adult bumblebees were caught and nests were divided into smaller parts to facilitate mite extraction. After that nests were placed on Tullgren funnels for 2 weeks in order to extract mites from nest material. Two weeks is a longer time than usually recommended for soil mites (Walter and Krantz 2009), but prolonged extraction is necessary as it takes up to 2 weeks for all bumblebees to emerge. The method with Tullgren funnels is suitable for extracting mites living in nest material, but inadequate for tracheal mites, and was chosen intentionally for this study. Extracted mites were preserved in 75% alcohol. All mites extracted from the nests were segregated under an Olympus SZ40 stereomicroscope. For species identification, mites were routinely mounted in Hoyer’s medium (Krantz 1978) on microscope slides, then counted and identified using an Olympus BX51 microscope and standard references (Micherdziński 1969; Hughes 1976; Hyatt 1980; Karg 1993). In the case of the most numerous astigmatid mite, the number of individuals per nest was counted in a Petri dish of 28.3 cm^2^ surface, with an overlain network of 1 cm^2^ squares. Counting was done with mites evenly spread on the Petri dish in water. After counting the number of mites in five squares, the number of individuals in the nest was extrapolated to the entire Petri dish surface.

Because colonies spent from 8 to 18 weeks in greenhouses, we examined if there is a relationship between the time the nest stayed in the greenhouse and mite number, using Spearman correlation. The independent variable was the time (weeks spent in the greenhouse), whereas the dependent variable was the number of mites from the two most abundant mite families in our samples (Acaridae and Laelapidae). The Parasitidae family was not considered because it was only occasionally encountered. A Mann–Whitney *U* test was used to compare differences in species composition between the suppliers.

### Commercial bumblebee nests before exploitation in greenhouses

Nests originated from two suppliers and were not used in greenhouses. They were investigated to assess if bumblebee colonies enter greenhouses already infested with mites. This part of the study was conducted on 20 bumblebee colonies purchased in 2007 (10 colonies, five from each supplier) and 2009 (the same sample sizes as in 2007) directly from two suppliers providing the nests to the greenhouses studied previously. Nests were placed on Tullgren funnels for 2 weeks. All procedures were the same as described above.

## Results

### Nests after exploitation in greenhouses

**Fig. 1:**
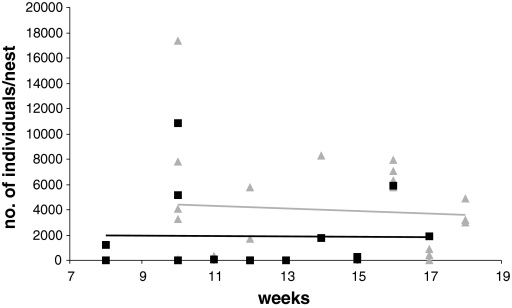
Correlation between nest age and number of Acaridae mites in two commercial greenhouses from southern Poland: Sarnów (*triangles* and *grey trend line*, R_s_ = 0.03, *p* = 0.90) and Krzeszowice (*squares* and *black trend line*, R_s_ = 0.18, *p* = 0.52)

In all 37 nests analyzed in 2006 we found seven mite species belonging to three families: Acaridae: *Tyrophagus putrescentiae* (Schrank); Laelapidae: *Hypoaspis marginepilosa* (Sellnick), *Hypoaspis hyatti* (Evans and Till), *Hypoaspis bombicolens* (Canestrini); Parasitidae: *Parasitellus fucorum* (De Geer), *Parasitellus ignotus* (Vitzthum), *Parasitellus crinitus* (Oudemans). We found no mite species foreign to the Polish or European fauna. The dominant species in both greenhouse complexes was a representative of the family Acaridae—*T. putrescentiae*. Differences in number of *T. putrescentiae* between suppliers were insignificant in both Sarnów and Krzeszowice (Table 1). The average number per nest in greenhouse complex Sarnów was 4,017 ± 894 (mean ± SE). In Krzeszowice this value amounted to 1,895 ± 801. The number of *T. putrescentiae* individuals per nest was independent of time spent in a greenhouse (Fig. 1).

**Table 1:** Number of mite species per nest of commercial bumblebees in Sarnów and Krzeszowice greenhouse complexes in southern Poland and parameters of the Mann–Whitney-*U* test for differences between suppliers

Sarnów							Krzeszowice					
	Supplier						Supplier					
	Israel (11)		The Netherlands (11)		*U* test		Israel (5)		The Netherlands (10)		*U* test	
Mite species	Mean	SE	Mean	SE	*p*	Z	Mean	SE	Mean	SE	*p*	Z
*Tyrophagus putrescentiae*	3,696.1	1,585.0	4,338.2	903	0.25	−1.15	3,569.2	2,039.5	1,058.7	581.7	0.29	1.05
*Hypoaspis marginepilosa*	28.4	8.3	1.5	0.5	0.004	2.85	41.4	20.0	31.0	10.0	0.58	0.55
*H. hyatti*	22.6	6.6	0.8	0.5	0.003	2.95	47.4	21.3	19.5	7.0	0.30	1.04
*H. bombicolens*	8.8	3.1	0.4	0.2	0.003	2.92	17.0	7.2	5.7	2.3	0.20	1.30
*Parasitellus fucorum*	0	0	0.1	0.1	0.72	−0.36	0.6	0.2	0.5	0.3	0.40	0.84
*P. ignotus*	0	0	0	0	–	–	0	0	0.3	0.1	0.18	−1.3
*P. crinitus*	0	0	0	0	–	–	0.2	0.2	0	0	0.16	1.4

The number in parentheses denotes the number of commercial nests from each supplier

**Fig. 2:**
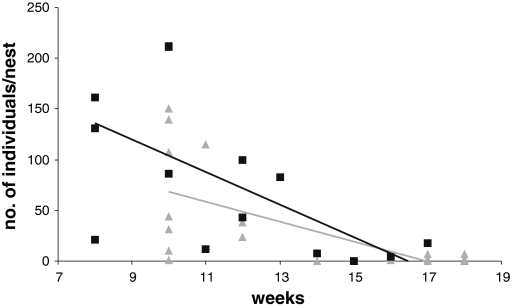
Correlation between nest age and number of Laelapidae mites in two commercial greenhouses from southern Poland: Sarnów (*triangles* and *grey trend line*, R_s_ = −0.62, *p* = 0.002) and Krzeszowice (*squares* and *black trend line*, R_s_ = −0.72, *p* = 0.002)

From the Laelapidae family, the most numerous was *H. marginepilosa*, then *H. hyatti,* whereas the least numerous was *H. bombicolens* (Table 1). Representatives of the Laelapidae family occur in mean number of 31 ± 10 (mean ± SE) individuals per nest in Sarnów and 73 ± 20 individuals in Krzeszowice. All three Laelapidae species differed in number of individuals between suppliers at the Sarnów locality, but did not differ in Krzeszowice (Table 1). The number of Laelapidae mites was significantly negatively correlated with time the nest spent in greenhouses at both studied localities (Fig. 2).

Representatives of the Parasitidae family were less numerous, we noted only 13 individuals in nests after greenhouse exposition. We refrained from conducting comparisons between suppliers and correlations with age of nest as these are not meaningful with such a low number of individuals. All *Parasitellus* were female deutonymphs, so they were at the phoretic stage. *Parasitellus fucorum* was represented by nine individuals found in seven commercial nests exposed for 10–16 weeks in greenhouses. *P. crinitus* was represented by only one specimen, detected in a nest exposed for 10 weeks. Three *P. ignotus* individuals were found in three nests, two of them spent 17 weeks in commercial greenhouse and one spent 8 weeks.

### Nests before exploitation in greenhouses

We did not find any mite species in nests studied in 2007 and 2009, purchased directly from suppliers.

## Discussion

To our knowledge this is the first study documenting the abundance and species composition of mites associated with nests of commercial bumblebees. We did not detect mite species foreign to the Polish fauna in bumblebee nests after greenhouse use. The mite species found in commercial bumblebee nests after greenhouse exploitation are common and widely distributed throughout Europe. Surprisingly, we did not find any mites in commercial nests before greenhouse exposition (despite sampling 2 years and two suppliers), thus bumblebee colonies probably enter greenhouses without nest-associated mites.

Our results indicate that nests directly from suppliers arrive in greenhouses free of mites as we did not detect mites during two independent sampling years (2007 and 2009). The time gap of 2 years between sampling in second experiment shows that our results are repeatable and therefore robust. Moreover, nests were randomly ordered within sampling seasons. Commercial bumblebee colonies are produced under well standardized conditions (Velthuis and Van Doorn 2006) implying that, in general, commercial nests from two investigated suppliers are mite free. Further questions pertain to the origin of mites in commercial bumblebee nests and the timing of infestation. Because the procedure for commercial bumblebee nest usage recommends the immediate placement of nests in greenhouses, it is most likely that they become infested with mites during this time.

The lack of mites in nests obtained directly from suppliers can be the result of rearing methods that curb mite infestation. The simplest way to control mite presence is to decrease humidity, as many species are not able to complete development at a relative humidity lower than 60% (Sánchez-Ramos et al. 2007; Eaton and Kells 2009). On the other hand, favorable developmental conditions for several mite species undoubtedly occur in greenhouses, from which we obtained nests after exploitation. Greenhouses are warm, humid and rich in organic matter from plants, soil and pollen, constituting ideal conditions for mite reproduction and development.

Among nest-inhabiting mites, we found the saprophagic and fungivorous species *T. putrescentiae*, and also mites that are saprophagous or predatory (families: Laelapidae and Parasitidae), depending on their instar. *T. putrescentiae* was the most common mite in the examined nests. This is a small species (~0.5 mm) frequently associated with a wide variety of stored food products, particularly those with a high content of fat, protein and moisture (Hughes 1976; Rodriguez and Rodriguez 1987; Hill 2002; Sánchez-Ramos and Castañera 2005; Canfield and Wrenn 2010). *T. putrescentiae* mainly feeds on fungi that develop on stored food products. In nests of bumblebees, the most appropriate food supply for this mite can be fungi developing on pollen, as this product is protein rich and easily becomes moist, forming conditions for mold development. Moreover, commercial bumblebee colonies are provided with honeybee pollen (Velthuis and Van Doorn 2006) on which 16 mite species were found, including *T. putrescentiae* and *P. fucorum* (Chmielewski 2003). Probably pollen supplies and high humidity in greenhouse and bumblebee nests promote the occurrence of *T. putrescentiae*. The observed high number of individuals can be explained by the short life cycle of this species—2 to 3 weeks from egg to adult stage under optimal conditions (23°C and relative humidity 87%, Hughes 1976). As there was no correlation between the time the nest was exploited and number of *T. putrescentiae*, we conclude that it can find proper conditions to live, reproduce and feed in other microhabitats inside greenhouses. A bumblebee nest may be one of many habitat types where *T. putrescentiae* finds sufficient organic matter, regardless of the developmental stage of the bumblebee colony.

The family Laelapidae, second in abundance in the studied nests, includes species that have varying degrees of association with other animals, both vertebrates and invertebrates (Mašán and Stanko 2005; Berghoff et al. 2009; Faraji and Halliday 2009). They have been previously recorded from rodent nests and wild bumblebee nests (Bregetova 1977) and are commonly found in Europe as predators feeding on smaller arthropods. Phoretic forms of *H. marginepilosa* were detected on foraging wild bumblebees in central Poland (Chmielewski and Baker 2008). They are associated with bees mainly because of transportation, but probably they also profit from living in their nests, feeding on honey and surface lipids of pollen provisions (Royce and Krantz 1989). Predatory instars can find prey among nest-inhabiting arthropods, mainly astigmatid mites (Costa 1966; Hunter and Husband 1973). We found a negative correlation of laelapid mite number and the time that a nest spent in a greenhouse. This can be a consequence of the synchronization between bumblebee and phoretic mite life cycles. Under natural conditions mites leave the nest attached to young bumblebee queens and overwinter with the insects. Most of the examined commercial nests harbouring laelapid mites were at the stage of the appearance of the sexual generation, or even after young queen and male production. As a result, the number of mites inhabiting bumblebee nests decreased over time. Nests considered in this study spent eight or more weeks in greenhouses, therefore the observed decline in mite numbers concerns only this period of time. Probably commercial nests are at first gradually inhabited by laelapid mites during greenhouse exploitation. After several weeks a decrease in number of mite-associates is observed as they leave bumblebee colonies attached to newly emerged queens and drones.

The family Parasitidae (genus *Parasitellus*) was less numerous in the studied material. Generally, representatives of Parasitidae can be found in moss, forest litter and grassland humus, decaying organic substrates and the nests of small mammals and insects (Micherdziński 1969; Bhattacharyya 1962, 1963; Hyatt 1980; Karg 1993; Mašán and Stanko 2005; Lindquist et al. 2009). All species of *Parasitellus* are obligatory associates of bumblebees in the Holarctic region, incidentally recorded from other insects, mammalian nests or nests of birds (Hyatt 1980). Under natural conditions, *P. fucorum* deutonymphs overwinter on young bumblebee queens. Adult females and deutonymphs feed mainly on pollen, wax, and nectar coating the pollen grains (Richards and Richards 1976; Schmid-Hempel 1998; Koulianos and Schwarz 1999). Pollen consumption by females and deutonymphs could have a negative effect on a bumblebee host if many mites occur in the nest. However, it may be advantageous for bumblebees to host oophagous and predatory protonymphs, as they reduce the abundance of other arthropods that can decrease colony condition due to depletion of pollen storage in nests (Schmid-Hempel 1998). In our study, it was the least abundant mite family, so the impact of these mites on bumblebees can be neglected in this case.

As there is a lack of information about commercial bumblebee associates, we are able to confront our results only with studies on wild bumblebees and their phoretic mites. Chmielewski and Baker (2008) found *P. fucorum* in 17% out of 425 specimens of wild bumblebees belonging to four bumblebee species: *B. lapidarius*, *B. lucorum*, *B. terrestris*, and *B. pascuorum*. They had up to ten deutonymphs on the thorax (Chmielewski and Baker 2008). In other studies performed in Switzerland, Schwarz et al. (1996) checked spring queens for phoretic mites and found the same five species as we did: *H. bombicolens*, *H. marginepilosa*, *H. hyatti*, *P. fucorum* and *P. ignotus*.

All species detected in our study also occur in the wild. One of these, *T. putrescentiae*, inhabits commercial bumblebee nests at high rates and this is a newly recorded microhabitat for this species, created by greenhouse agriculture and ecological pollination methods. *T. putrescentiae* is susceptible to humidity and temperature decrease (Sánchez-Ramos and Castañera 2005; Sánchez-Ramos et al. 2007) and a greenhouse environment ensures favorable conditions for its development. The other six species we noted are bumblebee associates, recorded in several studies on wild bumblebees and their phoretic mites. These species probably invaded greenhouses as instars, phoretic on bumblebees that escaped to forage outside the greenhouses and then returned (Whittington et al. 2004; Kraus et al. 2011). Parasitidae and Laelapidae representatives take advantage of the accessibility of food for pollen-feeding instars and for predatory ones. Probably some of these mites hibernate on bumblebees outside of greenhouses, but the rest die as the greenhouse season ends and food supplies are diminished both for bumblebees and mites.

The conducted experiments on commercial nests before and after greenhouse use permit a better understanding of mite species composition and abundance, but these results evoke new questions. Therefore further studies are needed to determine how mite species enter bumblebee nests, how quickly nests are occupied and by which species. We found that the number of Laelapidae mites in nests decreases with time spent in greenhouses; however this involves only nests which have spent eight or more weeks in greenhouses. We do not know anything about mite species and their numbers in nests that spent less time in greenhouses. Although we did not detect mites in commercial bumblebee nests before greenhouse usage, we advise at least occasional monitoring of commercial nests for the presence of mites. Currently such monitoring is not carried out; instead, bumblebees are checked for honeybee parasites (Velthuis and Van Doorn 2006)—an unreasonable procedure considering that bumblebees themselves host quite a variety of parasites (Goulson 2003). Some recent studies have shown that commercial bumblebee colonies impact the local bumblebee fauna by transfer of protozoan parasites (Colla et al. 2006; Otterstatter and Thomson 2008) and parasitic tracheal mites (Goka et al. 2001, 2006; Yoneda et al. 2008a, b). Our report on the lack of mites in nests imported directly from suppliers and, in consequence, the absence of foreign mite species in nests after greenhouse exploitation, are of considerable importance in the context of invasive species and their influence on local biodiversity. Our results argue for moderate optimism concerning the safety of commercial bumblebee use for pollination services, as they show that in the case of well-controlled conditions during the production of bumblebee nests, the spread of potentially harmful and foreign mite species is minimal.
